# Olf1/EBF associated zinc finger protein interfered with antinuclear antibody production in patients with systemic lupus erythematosus

**DOI:** 10.1186/ar2972

**Published:** 2010-04-01

**Authors:** Xuebing Feng, Rongliang Li, Jing Huang, Huayong Zhang, Lina Zhu, Bingzhu Hua, Betty P Tsao, Lingyun Sun

**Affiliations:** 1 Department of Rheumatology, Nanjing Drum Tower Hospital, the Affiliated Hospital of Nanjing University Medical School, 321 Zhongshan Road, Nanjing, 210008, PR China; 2 Division of Rheumatology, David Geffen School of Medicine at UCLA, 1000 Veteran Avenue, Los Angeles, CA 90095-1670, USA

## Abstract

**Introduction:**

The aim of the study was to determine whether Olf1/EBF associated zinc finger protein (OAZ), a transcription factor encoded by a positional systemic lupus erythematosus (SLE) candidate gene, plays a functional role in the pathogenesis in SLE.

**Methods:**

Gene expression levels in peripheral blood cells (PBLs) measured using quantitative real-time polymerase chain reaction (qPCR) were assessed for association with disease activity and the presence of specific autoantibodies. Peripheral blood mononuclear cells (PBMCs) were incubated with specific siRNAs for three days, then cells were harvested for measuring mRNA levels using qPCR, and supernatants for levels of total immunoglobulin (Ig)G and IgM as well as secreted cytokines, chemokine and antinuclear antibodies (ANA) using ELISA. Indirect immunofluorescence was also applied for ANA detection.

**Results:**

OAZ gene expressions in PBLs from 40 ANA-positive SLE patients were significantly increased than those from 30 normal controls (*P *< 0.0001) and 18 patients with rheumatoid arthritis (*P *< 0.01). In SLE patients, OAZ transcripts were positively correlated with SLE disease activity index (SLEDAI) score (r = 0.72, *P *< 0.0001) and higher in those positive for anti-dsDNA or anti-Sm antibodies (both *P *< 0.05). Co-culturing with OAZ siRNAs reduced mRNA levels of OAZ by 74.6 ± 6.4% as compared to those co-cultured with non-targeting siRNA and OAZ silencing resulted in reduced total IgG, ANA, interferon (IFN)-γ, interleukin (IL)-10, IL-12 and IL-21, but elevated CCL2 levels in culture supernatants (*P *< 0.05). The declined ANA levels correlated with inhibited OAZ expression (r = 0.88, *P *= 0.05), reduced IL-21 levels (r = 0.99, *P *< 0.01), and elevated chemokine (C-C motif) ligand 2 levels (r = -0.98, *P *< 0.01). Expressions of ID1-3 were significantly down-regulated by 68.7%, 70.2% and 67.7% respectively after OAZ silence, while ID3 was also highly expressed in SLE PBLs (*P *< 0.0001) and associated with disease activity (r = 0.76, *P *< 0.0001) as well as anti-dsDNA or anti-Sm antibodies (both *P *< 0.05).

**Conclusions:**

Elevated expression of OAZ transcripts in SLE PBLs were strongly correlated with disease activity. Suppression of OAZ expression inhibited downstream ID levels, and secretion of ANA and IL-21, implicating a role of OAZ pathway in the pathogenesis of SLE.

## Introduction

Systemic lupus erythematosus (SLE) is a prototype autoimmune disease with relatively strong genetic components that genome wide linkage scans performed in the past decade have identified chromosome 16p12.3 to q12.2 to be the second-strongest locus linked to SLE after HLA (6p22.3 to 6p21.1) [1-8]. A positional candidate gene, ITGAM located at 16p11.2, has been recently identified and validated as a lupus susceptibility gene by different groups [9-11]. However, fine mapping experiments of the chromosome 16p locus have shown multiple association signals clustering in several regions [8,12], suggesting more than one gene may be involved in the increased risk of SLE.

We have previously shown overall skewing of the transmission of D16S517 (a microsatellite marker) alleles and preferential transmission of one of its alleles from heterozygous parents to offspring affected with SLE of Chinese descent [13,14]. D16S517 is located within intron 5 of a gene named Olf1/EBF associated zinc finger protein (OAZ, also known as zinc finger protein 423; ZNF423) in 16q12. Genotyping of five SNPs within intron 4 and intron 5 of OAZ reveals preferential transmission of haplotypes containing SNPs and/or the SLE-associated D16S517 allele [14], suggesting OAZ may be a positional candidate gene within the 16q interval.

Currently there is no evidence indicating OAZ in the pathogenesis of SLE. OAZ is a transcriptional factor that binds to DNA through its zinc fingers, which acts as a bone morphogenetic protein (BMP) induced co-regulator of the Smad1 to Smad4 complex [15]. Through model biology study, OAZ is found to be necessary in BMP-4 induced gene activation [16]. Among the various genes activated via BMP signaling, ID (inhibitor of differentiation or inhibitor of DNA binding) proteins involved in the regulation of cell differentiation and proliferation may be the most important targets [17]. Four ID proteins (ID1 to 4) have been identified in mammals, in which ID3 is required for normal B cell functions [18]. Thus, it is plausible that OAZ may act through these IDs to regulate immune responses.

The presence of antinuclear antibodies (ANA), found in 95% of the patients, is the most prevalent feature of SLE that may play a pivotal role in the disease pathogenesis. These antibodies bind to a variety of macromolecules, including DNA, RNA and proteins, and some of them, notably anti-dsDNA and anti-Sm, are specific for the disease. Both the B-cell and T-cell compartments exhibit functional abnormalities that could lead to the autoantibody production in SLE [19]. Here we showed that OAZ expression levels were elevated in patients affected with SLE but not RA, correlated with disease activity and associated with positivity for anti-dsDNA and anti-RNA binding protein (anti-RBP) antibodies. In addition, silencing OAZ expression correlated with decreased production of ANA, IL-21, but elevated CCL2. These findings support a role of OAZ in the pathogenesis of SLE.

## Materials and methods

### Patients and controls

Study protocol was reviewed and approved by the Ethics Committee of the Affiliated Drum Tower Hospital of Nanjing University Medical School where all subjects were recruited, excluding those with a current infection. All patients and healthy volunteers provided written informed consents to participate in the study. Enrollment criteria for SLE patients were 1) fulfillment of the 1997 revised criteria of the American College of Rheumatology [20], and 2) with positive serum ANA (a titer >1:100 dilution) at the study entry. Healthy volunteers were recruited as normal controls with an effort to match age and gender of SLE patients. Rheumatoid arthritis (RA) patients who meet the 1987 American College of Rheumatology classification criteria [21] were recruited as disease controls. In total 40 SLE patients, 30 normal controls and 18 RA patients were enrolled for measuring the gene expressions in peripheral blood cells (Table 1).

**Table 1:** Demographics of SLE patients, normal controls and disease controls*

	SLE patients (n = 40)	Normal controls (n = 30)	Disease controls (n = 18)^+^
Age, years	33.48 ± 2.11 (14 to 70)	29.23 ± 1.13 (19 to 42)	38.39 ± 2.02 (18 to 54)
Female gender	33 (82.5%)	24 (80.0%)	17 (94.1%)
Disease duration, years	5.91 ± 0.94 (0.08 to 20)		
SLEDAI score	7.70 ± 0.90 (1 to 27)		
Autoantibodies			
Anti-dsDNA	15 (37.5%)		
Anti-Sm	19 (47.5%)		
Anti-SSA	18 (45.0%)		
Anti-SSB	14 (35.0%)		
Anti-U1RNP	9 (22.5%)		

* Values are shown by mean ± SEM (range) or number (percentage). There were no significant differences between patients with systemic lupus erythematosus (SLE) and normal controls or disease controls in terms of age and gender.

^+^Consisted of 18 patients with rheumatoid arthritis. SLEDAI, Systemic Lupus Erythematosus Disease Activity Index.

At the beginning of the study, each SLE patient was assessed for the presence of ANA and IgG antibodies to anti-dsDNA and anti-extractable nuclear antigen using reagents from Euroimmun AG (Lübeck, Germany) and disease activity at the time of blood drawn was assessed by their rheumatologists and verified by two of the authors (RL and BH) using the SLE disease activity index (SLEDAI) [22]. Among the 40 patients having their gene expressions measured in peripheral blood, 38 were treated with steroids (an average dose of 34.6 ± 2.6 mg per day of methylprednisolone (from 6 mg to 80 mg according to their disease activity)), 18 were taking hydrochloroquine, 5 were on cellcept or cyclosporin and 8 were on non-steroidal anti-inflammatory drug at the time of blood draw.

### Sample collection and RNA processing

A 2 to 3 ml sample of blood was collected in BD Vacutainer spray-coated K2EDTA Tubes (BD Biosciences, San Jose, CA, USA) and total RNA from whole cells was extracted immediately using Trizol (Invitrogen, Carlsbad, CA, USA). A 1 to 2 μg aliquot of total RNA was reverse transcribed into complementary DNA (cDNA) using Superscript II reverse transcriptase (Invitrogen, USA). All RNA and cDNA samples were stored at -70°C before use.

### Gene expression measuring

Primers were designed using the Primer Express 2.0 Software (Applied Biosystems, Foster City, CA, USA) according to the total mRNA sequences of OAZ (NM_015069), ID1 (NM_002165), ID2 (NM_002166), ID3 (NM_002167) and ID4 (NM_001546). Human ribosomal protein, large, P0 (RPLP0) was used as the housekeeping gene to normalize cellular RNA amounts. All the primers were synthesized by Takara Corp (Shanghai, China) and the sequences were shown in Table 2.

**Table 2:** Primers for real-time PCR

Gene	Forward	Reverse
OAZ	CTG CTC ACA GTG CCC TCA GAA G	ACT GTG CGT GCT GGC TCA TC
ID1	GTA AAC GTG CTG CTC TAC GAC ATG A	AGC TCC AAC TGA AGG TCC CTG A
ID2	TGT CAG CCT GCA TCA CCA GA	CCA CAC AGT GCT TTG CTG TCA
ID3	TCA GCT TAG CCA GGT GGA AAT C	GGC TGT CTG GAT GGG AAG GT
ID4	GAT CCT GCA GCA CGT TAT CGA CT	AAT GCT GTC GCC CTG CTT G
RPLP0	GTT TCA TTG TGG GAG CAG ACA	CAT GGT GTT CTT GCC CAT CA

PCR, polymerase chain reaction.

Experiments were performed in triplicate for each sample in 96-well plates using SYBR Green I Dye in the Applied Biosystems 7500 real-time PCR system. Reactions were performed in a 20 μl reaction volume and cycling times and temperatures were as follows: initial denaturation was carried out for 10 seconds at 95°C, followed by 45 cycles of denaturation at 95°C for 5 seconds and combined primer annealing/extension at 60°C for 34 seconds. Data were displayed using SDS software, version 2.0 (Applied Biosystems, USA). The ΔCT value was determined by subtracting the RPLP0 (the house keeping gene) CT value from the target gene CT value. Relative gene expressions were calculated as 2^-(ΔCT each-ΔCT mean) ^where ΔCT each = ΔCT value of each sample, ΔCT mean = mean ΔCT values of normal controls.

### RNA Interference

Accell siRNA was a novel form of short-interfering RNA that was absorbed directly by cells without the use of conventional delivery methods such as transfection reagents or viruses. Four pairs of siRNA sequences targeting OAZ were designed and synthesized by Dharmacon (Accell SMARTpool siRNA A-012907 13-16, Thermo Fisher Scientific, Waltham, MA, USA). SiRNA with a non-targeting sequence was used as a negative control, and siRNA targeting glyceraldehyde-3-phosphate dehydrogenase (GAPDH) was used as a positive control (Table 3).

**Table 3:** Accell siRNA sequences

Gene	Sense	Antisense
OAZ	GUA AUG AAU AUA AUC GGU U	AAC CGA UUA UAU UCA UUA C
OAZ	GCA UCA ACC ACG AGU GUA A	UUA CAC UCG UGG UUG AUG C
OAZ	GGA GGA UGA AUC AAU UUA C	GUA AAU UGA UUC AUC CUC C
OAZ	CCU UCA UGU AUU AUA UUG A	UCA AUA UAA UAC AUG AAG G
Nt	UUC UCC GAA CGU GUC ACG U	ACG UGA CAC GUU CGG AGA A
GAPDH	GUA UGA CAA CAG CCU CAA G	CUU GAG GCU GUU GUC AUA C

Nt, non-targeting siRNA

Blood was drawn from five randomly selected female SLE patients (40.40 ± 1.69 yrs old) and OAZ gene silencing was performed according to the manufacturer's instructions (Dharmacon). Briefly, mononuclear cells were collected by Ficoll-Hypaque discontinuous gradient, resuspended in siRNA buffer, distributed to 96-well plate at 2 × 10^5 ^per well, and then divided into OAZ silence group (*OAZ sil*, added with OAZ siRNA mixture using SMARTpool technique), positive control group (added with GAPDH siRNA), negative control group (*Neg ctl*, added with non-targeting siRNA) and mock group (only culture medium added). For each sample, 4 to 10 replications in each group were applied. Cells were incubated at 37°C with 5% CO_2 _for 72 hour and trypan blue exclusion test of cell viability was done by counting 200 cells before and after cell culture. After culturing, supernatants were collected and cells were harvested, RNA was extracted and then reverse transcribed to cDNA. Specific inhibition efficacy of OAZ, ID1-4 and GAPDH was confirmed by qPCR.

### Immunoglobulin and antinuclear antibody detection

Total IgG and IgM levels were measured by commercial ELISA kits (Westang Bio-Tech Co., Shanghai, China). Cultured supernatants were diluted in 1:10 for IgG test and undiluted for IgM test. ANA levels were detected by ELISA and also indirect immunofluorescence according to manufacturer's instruction (Euroimmun, Lübeck, Germany). An ANA value for each sample was calculated by OD _sample_/OD _standard _obtained from the ELISA test. As for the immunofluorescence test, undiluted samples were layered on HEp-2 cell spots and incubated for 30 minutes. After washing, fluorescein-labeled anti-human globulin was added to each spot and incubated for another 30 minutes. Slides were then embedded and read under the fluorescent microscope (Olympus; Tokyo, Japan). The criterion for assigning intensity of fluorescence was as follows: 4+ (very bright green), 3+ (bright green), 2 + (green), 1 + (faint green).

### Cytokine and chemokine measurements

Interferon γ (IFN-γ), interleukin 4 (IL-4), IL-10, IL-12, IL-21 and chemokine (C-C motif) ligand 2 (CCL2) levels in supernatants obtained from the previous step were measured by EIA kits (R & D Systems, Minneapolis, MN, USA). For each cytokine, samples and diluted standards were added to pre-embedded plate and incubated for two hours at 37°C. Then biotinylated anti-human detection antibody was added to each well. The plate was incubated for one hour at 37°C and developed with the addition of Streptavidin-HRP and tetramethylbenzidine as a substrate. The optical density for each well was documented with a microplate reader (Tecan Sunrise, Männedorf, Switzerland) set to 450 nm and levels of cytokines and chemokine (pg/ml) were calculated according to their standard curves.

### Statistical analysis

Results are presented as means ± SEM. Because gene expressions were extremely altered in certain patients, those levels were transformed to log10 values for analysis. To observe the changes of gene expressions, cytokine/chemokine levels and ANA values due to OAZ silence, an excitation-inhibition ratio (EIR) for each sample was calculated according to the formula: . For comparing the results between two groups, Student's t-test was conducted if the variance was normally distributed, whereas the Mann-Whitney U test was used if the variance was not normally distributed. Correlation between groups was evaluated using the Pearson rank test. Data were analyzed using the Prism 3.0 program (GraphPad, La Jolla, CA, USA), and *P *< 0.05 was considered significant.

## Results

### OAZ highly expressed in SLE patients and correlated with disease activity

**Figure 1:**
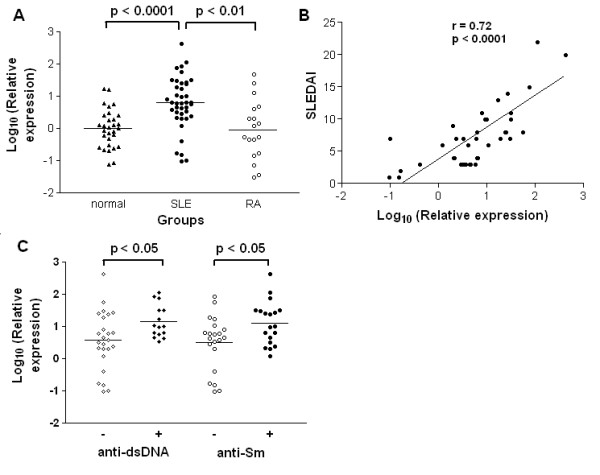
**OAZ highly expressed in SLE patients and associated with disease activity as well as anti-dsDNA and anti-Sm antibodies**. **a**. Elevated OAZ expressions in peripheral blood cells from SLE patients. Each symbol represents an individual sample and horizontal lines shows mean values. The log10 values of OAZ expression levels were 0.79 ± 0.13 in SLE patients, 0.001 ± 0.109 in normal controls and -0.04 ± 0.22 in RA patients. **b**. OAZ expression correlated with SLE disease activity index (SLEDAI). According to Pearson's correlation test, the log10 values of OAZ expression levels in peripheral blood cells from 40 SLE patients were significantly correlated with SLEDAI scores (r = 0.72, *P *< 0.0001). **c**. OAZ expression associated with anti-dsDNA and anti-Sm antibodies. Each symbol represents an individual sample and horizontal lines showed mean values. Patients were divided into two groups according to their anti-dsDNA or anti-Sm antibody detection results at the time of blood drawn. Those with positive anti-dsDNA antibody or anti-Sm antibody had a higher expression of OAZ in peripheral blood than those without (both *P *< 0.05).

To explore whether OAZ was differentially expressed in SLE, mRNA levels relative to a house keeping gene were measured by qPCR using peripheral blood cells from 40 SLE patients, 30 matched normal controls and 18 RA patients, and then transformed to log10 values. As shown in Figure 1a, OAZ gene were highly expressed in peripheral blood samples from SLE patients as compared to those from normal controls (0.79 ± 0.13 vs 0.001 ± 0.109, *P *< 0.0001) and RA patients (0.79 ± 0.13 vs -0.04 ± 0.22, *P *< 0.01), while there was no difference between those from normal controls and RA patients (*P *> 0.05). The expression data of OAZ in SLE patients were compared with varying levels of disease activity as assessed by the SLEDAI score at the time blood was obtained. A positive correlation between OAZ values and SLEDAI scores was observed (Pearson r = 0.72, *P *< 0.0001) (Figure 1b).

### OAZ associated with autoantibody production in SLE patients

Since the production of antinuclear autoantibodies is the most ubiquitous feature in SLE, SLE predisposing factors should impact on ANA production [23]. Within our 40 patients, all had a positive ANA (titers >1:100) at the time of blood draw, while 15 had concurrent IgG anti-dsDNA, 19 had anti-Sm, 18 had anti-SSA, 14 had anti-SSB and 9 had anti-U1 RNP. We found OAZ expressions were higher in patients with positive anti-dsDNA antibodies as well as in those with positive anti-Sm antibodies (both *P *< 0.05, Figure 1c). However, there were no associations between OAZ expression and anti-SSA, SSB or U1RNP antibodies (data not shown).

### Diminished ANA production after OAZ silence

**Figure 2:**
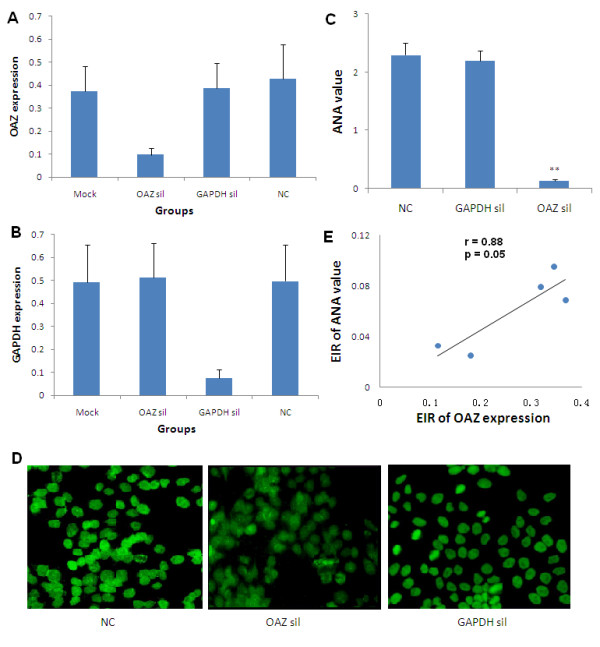
**Blockade of ANA production after OAZ silencing in vitro**. **a**. OAZ expression decreased after the silence of OAZ. The highest expression level was set to be 1 (100%) and expression data in Mock group (mock), OAZ silence group (OAZ sil), GAPDH silence group (GAPDH sil) and negative control group (NC) were shown as mean values ± SEM. A significant decline of mRNA expression was found in the OAZ silence group comparing to that in the negative control group (*P *< 0.05). **b**. GAPDH expression dramatically decreased after the silence of GAPDH. Values were shown as mean ± SEM. A significant decline of mRNA expression was found in the GAPDH silence group comparing to that in the negative control group (*P *< 0.05). **c**. Decreased ANA level after OAZ silencing. ELISA was preformed to measure the production of ANA and data were shown as mean OD values ± SEM. A dramatically decline of ANA production was found in the OAZ silence group as compared to negative control group and GAPDH silence group (** *P *< 0.01). **d**. Decreased intensity of ANA fluorescence after OAZ silencing. Representative photomicrographs of ANA fluorescence in negative control group, OAZ silence group and GAPDH silence group were shown. After OAZ silence, only weak fluorescence was observed. **e**. Association of OAZ expression and ANA level. The excitation-inhibition ratio (EIR) of OAZ expression was positively correlated with EIR of ANA value after gene silencing (Pearson r = 0.88, *P *= 0.05).

To assess whether OAZ expression might regulate autoantibody production in vitro, mRNA expression in peripheral blood mononuclear cells from SLE patients were knocked down by OAZ siRNA, with siRNA targeting GAPDH as a specificity control, or siRNA with a non-targeting sequence as a negative control. The percentages of viable cells were 97.6 ± 1.5% before cell culture and 94.7 ± 1.3% after the culture and there were no differences in viability among groups. Compared to those from the negative control group (using non-targeting siRNA), OAZ expressions were significantly decreased only in culture cells treated with siRNA specific for OAZ with a 74.6 ± 6.4% reduction of expression level (Figure 2a). Mean expression of GAPDH was not inhibited by OAZ siRNA, and was decreased by 84.9 ± 4.7% by GAPDH siRNA in the positive control group (Figure 2b).

ANA were present in all studied SLE patients, making ANA the most useful test to measure the change of autoantibodies after OAZ knockdown. Because most SLE patients did not have positive anti-dsDNA antibody and anti-Sm antibody in their serum, we measured ANA in culture supernatants as an alternative. As shown in Figure 2c, ANA value was 2.30 ± 0.20 in the negative control group and 0.13 ± 0.03 in the OAZ knockdown group measured by ELISA (*P *< 0.01). There was no difference in ANA value between negative control group and GAPDH knockdown group (*P *> 0.05). Consistent with ELISA results, intensity of ANA fluorescence was decreased from 2 to approximately 3+ in the control group to 1+ in OAZ silenced group assayed using indirect immunofluorescence (Figure 2d). There was a positive correlation between the EIR of OAZ expression and ANA value (r = 0.88, *P *= 0.05) (Figure 2e), suggesting that OAZ might play a role in ANA production.

To further confirm a relationship between OAZ expression and antibody production, total IgG and IgM in culture supernatants were measured. IgG levels were significantly decreased in the OAZ knockdown group as compared to those in the negative control group (26.8 ± 3.7 ug/ml vs 44.3 ± 6.4 ug/ml, *P *< 0.05), while there was no difference in IgM levels between the two groups (4.13 ± 0.56 ug/ml vs 2.82 ± 0.36 ug/ml, *P *= 0.09).

### Alteration of cytokine and chemokine levels after OAZ silence

**Figure 3:**
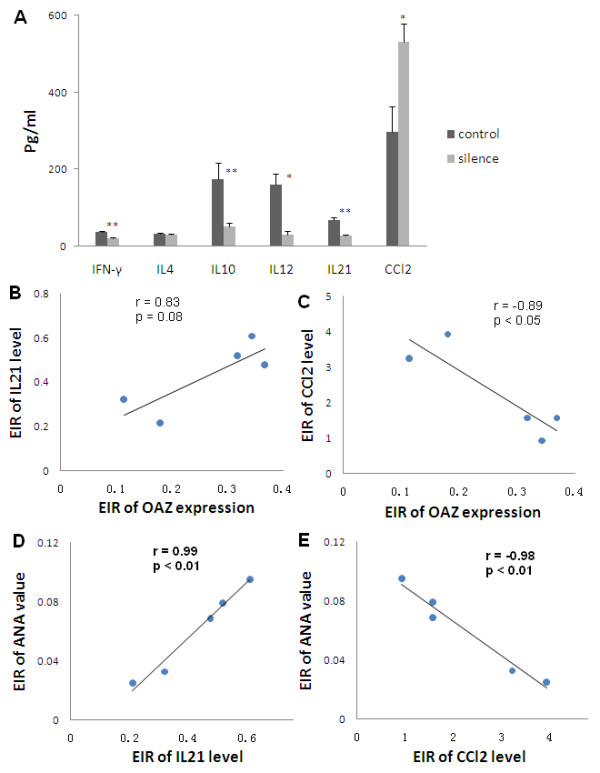
**Alteration of cytokine and chemokine levels after OAZ silencing**. **a**. IFN-γ, IL-10, IL-12 and IL-21 levels were significantly decreased in culture supernatants of OAZ silence group (silence) as compared to those of negative control group (control), while IL-4 levels remained unchanged and CCL2 levels were increased in culture supernatants of silence group. Values were shown as means ± SEM (** *P *< 0.01, * *P *< 0.05). **b, c**. The relationship between OAZ expression and IL-21 as well as CCL2. There was a tendency for the excitation-inhibition ratio (EIR) of OAZ expression to be positively correlated with EIR of IL-21 level (Pearson r = 0.83, *P *= 0.08), while the EIR of OAZ expression was negatively correlated with EIR of CCL2 level (Pearson r = -0.89, *P *< 0.05). **d, e**. Association of IL-21, CCL2 and ANA level. EIR of IL-21 level was positively correlated with EIR of ANA value (Pearson r = 0.99, *P *< 0.01), while EIR of the CCL2 level was negatively correlated with EIR of ANA value (Pearson r = -0.98, *P *< 0.01).

Production of IgG autoantibodies requires T cell help, and there are several subpopulations of effector CD4^+ ^T lymphocytes (Th cells) based on the cytokines they produced. To assess whether OAZ was linked to the cytokine disturbance that was a prominent phenomenon in SLE, we tested the secretion levels of two Th1 cytokines (IFN-γ and IL-12), two Th2 cytokines (IL-4 and IL-10) and one newly discovered Th17-related cytokine (IL-21). As shown in Figure 3a, four of the five cytokines measured were significantly down-regulated after OAZ silencing compared to those in the negative control group. The levels of IFN-γ, IL-10, IL-12 and IL-21 were 37.8 ± 2.6 pg/ml, 175.7 ± 42.0 pg/ml, 160.8 ± 28.1 pg/ml and 69.0 ± 5.5 pg/ml in the group treated with siRNA to OAZ, while the levels were 21.7 ± 1.5 pg/ml, 52.4 ± 8.9 pg/ml, 30.0 ± 9.3 pg/ml and 28.1 ± 3.6 pg/ml in the negative control group (*P *< 0.01, *P *< 0.01, *P *< 0.05 and *P *< 0.01 respectively). There was no difference inIL-4 level between the two groups. CCL2, a chemokine that has been implicated as a biomarker for lupus nephritis [24], was significantly increased in the OAZ silenced group compared to those in the negative control group (298.3 ± 65.3 vs 531.5 ± 46.5, *P *< 0.05) (Figure 3a).

To identify the relationship between OAZ expression and specific cytokines and/or chemokine, EIR was calculated as described before. There was a positive trend of correlation between the EIR of OAZ expression and the EIR of IL-21 level (r = 0.83, *P *= 0.08, Figure 3b), suggesting the more decreased levels of OAZ, the lower levels of IL-21. The EIR of OAZ expression was negatively correlated with the EIR of CCL2 level (r = -0.89, *P *< 0.05, Figure 3c), indicating a strong negative correlation between OAZ transcripts and the secreted levels of CCL2 in cultures of peripheral blood cells from SLE patients.

Strikingly, there was a perfect positive correlation between EIR of IL-21 levels and EIR of ANA value, and a negative correlation between EIR of CCL2 levels and EIR of ANA value (r = 0.99 and r = -0.98, both *P *< 0.01) (Figure 3d, e), providing evidence that these two factors might be important in the production of ANA.

### ID3 gene participated in OAZ pathway in SLE

**Figure 4:**
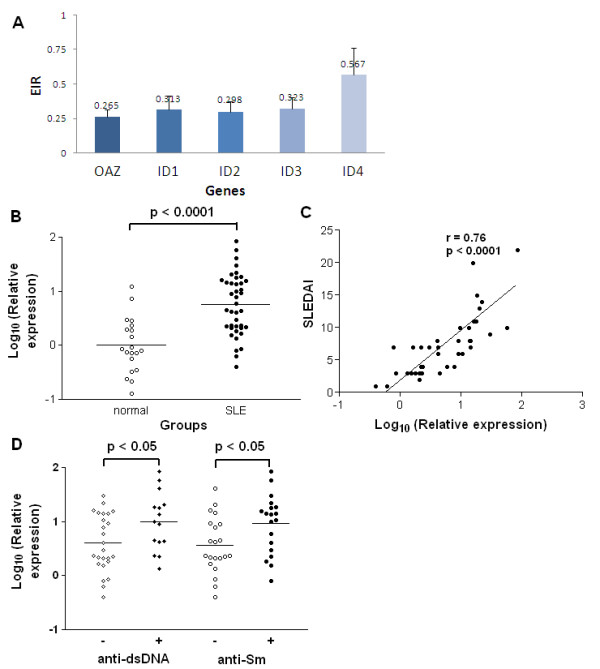
**ID3 associated with disease activity as well as anti-dsDNA and anti-Sm antibodies**. **a**. Expressions of ID genes decreased after OAZ silencing in vitro. Cells collected from silence study were applied for the detection of ID1 to 4 expressions using qPCR. The EIR was obtained for each subject and the inhibition efficacy was then calculated as (1-EIR) ×100%. After OAZ silencing, expression levels of ID1, ID2, ID3 and ID4 were reduced by 68.7%, 70.2%, 67.7% and 43.3%, respectively. **b**. Elevated ID3 expressions in peripheral blood cells from SLE patients. Each symbol represents an individual sample and horizontal lines show median values. The relative expression levels of ID3 were 0.75 ± 0.09 in SLE patients and 0 ± 0.11 in normal controls (*P *< 0.0001). **c**. ID3 expression correlated with SLE disease activity. According to Pearson's correlation test, log 10 values of ID3 expression levels were significantly correlated with SLEDAI scores (r = 0.76, *P *< 0.0001). **d**. ID3 expression associated with anti-dsDNA antibody and anti-Sm antibody. Each symbol represents an individual sample. Those with positive anti-dsDNA antibody had higher expression of ID3 in peripheral blood than those without (*P *< 0.05), as did those with positive anti-Sm antibody (*P *< 0.05).

Since ID expression might be regulated by OAZ, expressions of all the four ID genes were measured after OAZ knockdown. ID1 to 3, but not ID4, were significantly down-regulated with an average decline of 68.7%, 70.2% and 67.7%, respectively, as compared to those of negative control (Figure 4a), supporting that expression of these genes in SLE patients might be regulated by the pathway involving OAZ.

Among the four IDs, ID3 that had putative immunofunctions [25] was selected as a representative for *in vivo *studies. To assess whether ID3 expressions were related to the disease status, qPCR was applied using peripheral blood cells from 40 SLE patients and 20 normal controls. Our data showed that ID3 was highly expressed in SLE patients compared to that in normal controls (0.75 ± 0.09 vs 0 ± 0.11, *P *< 0.0001) and there was a positive correlation between ID3 expression level and SLEDAI score (r = 0.76, *P *< 0.0001) (Figure 4b, c). Meanwhile, a correlation between OAZ and ID3 expression levels was observed (r = 0.55, *P *= 0.0002). ID3 expressions were also elevated in those with positive anti-dsDNA or anti-Sm antibodies (Figure 4d), indicating that ID3 could play a role in the regulation of OAZ driven ANA production.

Abnormal activation of the type I interferon pathway presents in most SLE patients particularly in those expressing anti-RBP antibodies [26]. To address whether siRNA for OAZ might non-specifically inactivate the type I interferon pathway, the expression level of LY6E, a type I interferon inducible gene [27], was measured after OAZ knockdown. No significant alteration of LY6E expression was observed among groups treated with non-targeting GAPDH- or OAZ- targeting SiRNA (data not shown).

## Discussion

To our knowledge, this is the first report to implicate a functional role of OAZ, a novel positional lupus candidate gene, in the pathogenesis of SLE. Dysregulation of OAZ expression modulated the ANA production of SLE patients. Our studies showed that OAZ mRNA levels in peripheral blood cells were elevated in SLE patients as compared to those in either normal or disease controls (RA patients). Transcript levels of OAZ were highly correlated with disease activity (r = 0.72, *P *< 0.0001) as well as associated with the presence of autoantibodies to dsDNA or Sm (Figure 1). OAZ expressions were negatively correlated with methylprednisolone doses (data not shown), implying that there should be an even greater elevation of OAZ expression in those patients with high SLEDAI scores because they were treated with high dose of steroids. When OAZ expression in peripheral blood mononuclear cells was silenced in vitro, mRNA levels of OAZ and ID1-3 were concomitantly decreased, together with a reduction of IFN-γ, IL-10, IL-12 and IL-21 levels, an elevation of CCL2 level, and decreased levels of total IgG and ANA (Figures 2 and 3). The extent of decreased ANA level was correlated with the inhibition of OAZ expression, the reduction of IL-21 level and the elevation of CCL2 level. Interestingly, ID3, a gene that was down-regulated after OAZ silence, was also highly expressed in SLE patients and associated with disease activity as well as the presence of anti-dsDNA or anti-Sm autoantibodies (Figure 4).

OAZ is present in cell nucleus and acts as a DNA-binding partner of BMP-Activated Smads [15]. According to our results, mRNA expression of OAZ is very low in peripheral blood cells (approximately 0.002% of the expression level of the house-keeping gene), making it difficult to be detected in the protein level. To ensure a specific amplification of this gene, primers have been blasted after the designation [28]. Besides, only OAZ but not EHZF, an OAZ homology gene that highly expressed in primitive human hematopoietic cells [29], showed differential expression in bone marrow cells between SLE patients and healthy donors in our preliminary study. The Accell siRNA delivery method, which has recently been widely used to silence genes in many cell types including those *difficult-to-transfect *immune cells [30-32], provides us a highly efficient way to study genes with low expressions but important functions.

In this paper, OAZ was found to be associated with autoantibody production *in vivo *and *in vitro*, suggesting that highly expressed OAZ in SLE patients might promote differentiation of B lymphocytes to antibody-secreting cells. Support evidence for this hypothesis includes that 1) overexpression of OAZ in the neuroblastoma cells leads to enhanced differentiation [33], and 2) suppression of OAZ expression reduces cell growth and contributes to the blast crisis stage in chronic myelogenous leukemia mouse model as the result of the differentiation block [34]. However, it is not clear how OAZ mediates its role in cell differentiation at present. As one of the largest members of the Kruppel-like zinc finger protein family, OAZ provides specificity in BMP signaling while ID genes are thought to be important targets of BMP signaling [15,35]. Based on the study of osteoblast differentiation of mesenchymal stem cells, both RNA interference-mediated knockdown and constitutive overexpression of Id genes are associated with diminished cell differentiation, implicating that Id proteins could play a dual role in BMP-mediated differentiation [36].

Among the four ID proteins, ID3 is known to have important functions in the immune system, although its specific role is yet to be delineated. Young ID3-deficient mice (6 to 12 weeks old) have defective immune responses displaying as a B-cell defect in the proliferation and attenuated antibody production [25]. Meanwhile, the production of IgG2a (a Th1 response immunoglobulin) is impaired along with a decreased expression of IFN-γ when stimulated with anti-CD3 in these mice. When E2A, an ID3 antagonist gene, was knocked down, mice frequently developed ANA and proteinuria at age eight months or older [37]. Thus a sustained high expression of ID3 might trigger abnormal immunity in SLE patients, facilitating the production of autoantibodies. To support this notion, our current data have shown a good correlation between ID3 and OAZ expressions and an association of ID3 expression with both anti-dsDNA and anti-Sm antibodies. Besides ID pathway, OAZ has been shown to be a component of retinoic acid signaling, and plays a key role in retinoic acid-induced differentiation through the regulation of retinoic acid receptors [33]. In addition, OAZ may act as a corepressor of EBF (early B cell factor) [38], and thus inhibit the transcription of a lot B lineage genes including Pax-5 [39] and consequently affect plasma cell differentiation [40,41]. It is reasonable that these pathways could also be helpful in OAZ induced B lymphocyte differentiation and antibody production in SLE patients.

Studies have implicated that cytokines and chemokines are involved in the differentiation of B lymphocytes into plasma cells [42,43]. The decreased level of Th1cytokines (IFN-γ and IL-12) in our case after OAZ silence is similar to that found in ID3-deficient mice [25], supporting a role of OAZ-ID3 pathway in this process. IL-10 is a well-known mediator of human B cell differentiation that has a powerful effect on the stimulation of immunoglobulin secretion by B cells in SLE [44]. IL-21 has a critical role in terminal B cell differentiation to plasma cells and has recently been identified as an important component in the development of autoimmune disease [45-47]. Our *in vitro *results suggested that OAZ could regulate IL-10 and IL-21 level in SLE. The near perfect correlation between the extent of IL-21 and ANA suppression by OAZ knockdown suggested that this cytokine could be a key mediator for OAZ induced B cell differentiation and ANA production. In addition to these cytokines, a chemokine, CCL2, has been shown to suppress plasma cell immunoglobulin production via STAT3 inactivation and PAX5 induction [48]. Of note, the expression of CCL2 is effectively regulated by BMP members [49]. In our experiment, CCL2 might work as a negative regulator of plasma cell differentiation when upregulated via OAZ silence.

## Conclusions

OAZ has been previously considered as a positional candidate gene for susceptibility to SLE. Our data provide evidence for functional relevance of OAZ in SLE in the production of antinuclear antibody, a hallmark of SLE, along with the alterations of several cytokines and chemokine especially those involved in the regulation of plasma cell function. As a complex network, BMP-Smad-IDs, retinoic acid receptor and Olf-1/EBF pathways could participate in OAZ-driven ANA production. Further studies are required for elucidating the molecular mechanisms of the OAZ gene in the development of autoimmune diseases.

## Abbreviations

ANA: antinuclear antibodies; Anti-RBP: anti-RNA binding protein; CCL2: chemokine (C-C motif) ligand 2; cDNA: complementary DNA; EBF: early B cell factor; EIR: excitation-inhibition ratio; GAPDH: glyceraldehyde-3-phosphate dehydrogenase; ID: inhibitor of differentiation/inhibitor of DNA binding; IFN: interferon; Ig: immunoglobulin; IL: interleukin; LY6E: lymphocyte antigen 6 complex, locus E; OAZ: olf1/EBF associated zinc finger protein; PBLs: peripheral blood cells; PBMCs: peripheral blood mononuclear cells; qPCR: quantitative real-time PCR; RA: rheumatoid arthritis; RPLP0: human ribosomal protein, large, P0; SLE: systemic lupus erythematosus; SLEDAI: SLE disease activity index.

## Competing interests

The authors declare that they have no competing interests.

## Authors' contributions

XF contributed to the design of the study, served as the study coordinator and drafted the manuscript. RL carried out the siRNA studies and cytokine/chemokine/ANA measurements. JH participated in the gene expression studies. HZ participated in data analysis. LZ and BH contributed to patient recruitment and management and to data collection. BPT was involved in critically revising the manuscript. LS participated in the design of the study and gave final approval of the version to be published. All authors read and approved the final manuscript.
